# 458. A Machine Learning Approach Identifies Distinct Early-Symptom Cluster Phenotypes Which Correlate with Severe SARS-CoV-2 Outcomes

**DOI:** 10.1093/ofid/ofab466.657

**Published:** 2021-12-04

**Authors:** Nusrat J Epsi, John H Powers, David A Lindholm, David A Lindholm, Alison Helfrich, Nikhil Huprikar, Anuradha Ganesan, Tahaniyat Lalani, Rupal Mody, Cristian Madar, Samantha Bazan, Rhonda E Colombo, Derek Larson, Ryan C Maves, Ryan C Maves, Gregory Utz, David Tribble, Brian Agan, Timothy Burgess, Allison Malloy, Simon Pollett, Stephanie A Richard

**Affiliations:** 1 HJF, Bethesda, Maryland; 2 Support to National Institute of Allergy and Infectious Disease, Bethesda, MD; 3 Uniformed Services University of the Health Sciences; Brooke Army Medical Center, San Antonio, TX; 4 Walter Reed National Military Medical Center, Bethesda, Maryland; 5 Walter Reed National Military Medical Center (WRNMMC), Bethesda, Maryland; 6 Infectious Disease Clinical Research Program and the Henry M. Jackson Foundation for the Advancement of Military Medicine and Walter Reed National Military Medical Center, Bethesda, MD; 7 Infectious Disease Clinical Research Program, Bethesda, MD, The Henry M. Jackson Foundation, Bethesda, MD, and Naval Medical Center Portsmouth, VA, Portsmouth, Virginia; 8 William Beaumont Army Medical Center, El Paso, Texas; 9 Tripler Army Medical Center, Tripler Army Medical Center, Hawaii; 10 Carl R. Darnall Army Medical Center, Fort Hood, Texas; 11 Madigan Army Medical Center, Tacoma, WA, Infectious Disease Clinical Research Program, Bethesda, MD, and Henry M. Jackson Foundation for the Advancement of Military Medicine, Inc., Bethesda, MD, Tacoma, Washington; 12 Fort Belvoir Community Hospital Infectious Disease, Fort Belvoir, Virginia; 13 Naval Medical Center San Diego, San Diego, CA and Infectious Disease Clinical Research Program, Bethesda, MD, San DIego, California; 14 Uniformed Services University of the Health Sciences, Bethesda, Maryland; 15 Uniformed Services University, Bethesda, MD; 16 Infectious Disease Clinical Research Program, USU/HJF, Bethesda, Maryland; 17 Infectious Disease Clinical Research Program, Bethesda, Maryland; 18 Infectious Disease Clinical Research Program, Department of Preventive Medicine and Biostatistics, Uniformed Services University of the Health Sciences, Bethesda, MD and Henry M. Jackson Foundation, Bethesda, MD, Bethesda, Maryland

## Abstract

**Background:**

The novel coronavirus disease 2019 (COVID-19) pandemic remains a global challenge. Accurate COVID-19 prognosis remains an important aspect of clinical management. While many prognostic systems have been proposed, most are derived from analyses of individual symptoms or biomarkers. Here, we take a machine learning approach to first identify discrete clusters of early stage-symptoms which may delineate groups with distinct symptom phenotypes. We then sought to identify whether these groups correlate with subsequent disease severity.

**Methods:**

The Epidemiology, Immunology, and Clinical Characteristics of Emerging Infectious Diseases with Pandemic Potential (EPICC) study is a longitudinal cohort study with data and biospecimens collected from nine military treatment facilities over 1 year of follow-up. Demographic and clinical characteristics were measured with interviews and electronic medical record review. Early symptoms by organ-domain were measured by FLU-PRO-plus surveys collected for 14 days post-enrollment, with surveys completed a median 14.5 (Interquartile Range, IQR = 13) days post-symptom onset. Using these FLU-PRO-plus responses, we applied principal component analysis followed by unsupervised machine learning algorithm k-means to identify groups with distinct clusters of symptoms. We then fit multivariate logistic regression models to determine how these early-symptom clusters correlated with hospitalization risk after controlling for age, sex, race, and obesity.

**Results:**

Using SARS-CoV-2 positive participants (n = 1137) from the EPICC cohort (Figure 1), we transformed reported symptoms into domains and identified three groups of participants with distinct clusters of symptoms. Logistic regression demonstrated that cluster-2 was associated with an approximately three-fold increased odds [3.01 (95% CI: 2-4.52); *P* < 0.001] of hospitalization which remained significant after controlling for other factors [2.97 (95% CI: 1.88-4.69); *P* < 0.001].

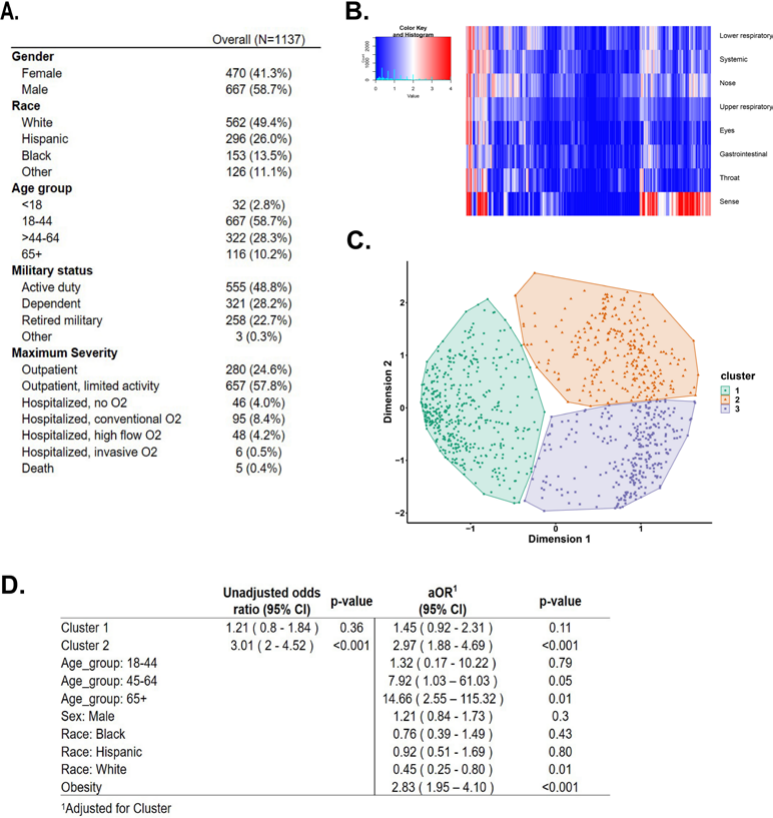

(A) Baseline characteristics of SARS-CoV-2 positive participants. (B) Heatmap comparing FLU-PRO response in each participant. (C) Principal component analysis followed by k-means clustering identified three groups of participants. (D) Crude and adjusted association of identified cluster with hospitalization.

**Conclusion:**

Our findings have identified three distinct groups with early-symptom phenotypes. With further validation of the clusters’ significance, this tool could be used to improve COVID-19 prognosis in a precision medicine framework and may assist in patient triaging and clinical decision-making.

**Disclaimer:**

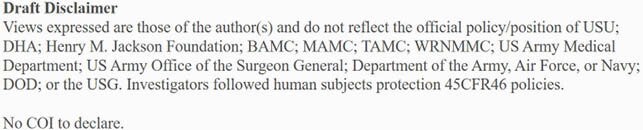

**Disclosures:**

**David A. Lindholm, MD**, American Board of Internal Medicine (Individual(s) Involved: Self): Member of Auxiliary R&D Infectious Disease Item-Writer Task Force. No financial support received. No exam questions will be disclosed ., Other Financial or Material Support **Ryan C. Maves, MD**, **EMD Serono** (Advisor or Review Panel member)**Heron Therapeutics** (Advisor or Review Panel member) **Simon Pollett, MBBS**, **Astra Zeneca** (Other Financial or Material Support, HJF, in support of USU IDCRP, funded under a CRADA to augment the conduct of an unrelated Phase III COVID-19 vaccine trial sponsored by AstraZeneca as part of USG response (unrelated work))

